# Establishing Pancreatic Cancer Organoids from EUS-Guided Fine-Needle Biopsy Specimens

**DOI:** 10.3390/cancers17040692

**Published:** 2025-02-18

**Authors:** Mei-Juan Wang, Chao Gao, Xin Huang, Min Wang, Shuai Zhang, Xiao-Pei Gao, Chang-Qing Zhong, Lian-Yong Li

**Affiliations:** Department of Gastroenterology, The Ninth Medical Center of Chinese PLA General Hospital, Beijing 100101, China; 18834564242@163.com (M.-J.W.); 13693067371@163.com (C.G.); huangxinlvxudan@163.com (X.H.); happywmin@126.com (M.W.); edwardeffi@163.com (S.Z.); shmily1886@126.com (X.-P.G.); zcq123456789@126.com (C.-Q.Z.)

**Keywords:** pancreatic cancer, organoids, EUS-FNA/FNB

## Abstract

Pancreatic cancer is a highly malignant digestive system tumor characterized by covert onset and rapid progression, with a 5-year survival rate of less than 10%. Most patients have already reached an advanced or metastatic stage at the time of diagnosis. Therefore, it is particularly important to study the occurrence, development, and drug resistance mechanisms of pancreatic cancer. In recent years, the development of 3D tumor cell culture technology has provided new avenues for pancreatic cancer research. Patient-derived organoids (PDOs) are micro-organ structures that are obtained directly from the patient’s body and rapidly expand in vitro. PDOs have the ability to self-renew and self-organize and retain the genetic heterogeneity and molecular characteristics of the original tumor. However, the use of organoids is limited because most patients with pancreatic ductal adenocarcinoma (PDAC) are inoperable. Endoscopic ultrasound-guided fine-needle aspiration/biopsy (EUS-FNA/FNB) is an important method for obtaining tissue samples from non-surgical pancreatic cancer patients. This article reviews the factors that affect the formation of pancreatic cancer organoids using EUS-FNA/FNB. High-quality samples, sterile operations, and optimized culture media are key to successfully generating organoids. Additionally, individual patient differences and disease stages can impact the formation of organoids. Pancreatic cancer organoids constructed using EUS-FNA/FNB have significant potential, suggesting new approaches for research and treatment.

## Simple Summary

This review addresses the urgent need to understand pancreatic cancer, a highly lethal disease with a 5-year survival rate below 10%, often diagnosed at advanced stages. The authors aim to leverage 3D tumor cell culture technology, specifically patient-derived organoids (PDOs), which replicate the genetic diversity and molecular traits of the original tumor, offering a novel approach to study cancer development and drug resistance. However, the inoperability of most pancreatic cancer patients poses a challenge. This research focuses on endoscopic ultrasound-guided fine-needle aspiration/biopsy (EUS-FNA/FNB) as a method to obtain samples for PDO creation. The findings emphasize the importance of sample quality, sterile procedures, and optimized media in organoid generation, acknowledging patient variability and disease stage as influential factors. The study’s results could revolutionize pancreatic cancer research by providing a viable model for studying disease mechanisms and developing personalized treatments, potentially improving patient outcomes and transforming clinical practices.

## 1. Introduction

Pancreatic cancer is a malignant tumor of the digestive system characterized by covert onset but rapid progression, very poor treatment outcomes and prognosis, and a 5-year survival rate of less than 10% [1]. Despite the continuous development of various diagnostic technologies in recent years, the early diagnosis rate of pancreatic cancer remains less than 5%, with 60% of pancreatic cancer patients already in the metastasis stage and 30% of patients in the locally advanced stage upon diagnosis [2]. Overall, 90% of pancreatic cancers are pathologically classified as pancreatic ductal adenocarcinoma (PDAC). The high mortality and increasing incidence of PDAC require a better understanding of its pathophysiology, pathogenesis, and drug resistance characteristics. In vitro models play important roles in research due to the inability of transformed cell lines to recapitulate the complexity of natural organs and the developmental, genetic, and physiological differences that limit the effectiveness of animal models [3]. Patient-derived xenografts (PDXs) are developed by directly implanting resected tumors from patients into immunodeficient animal models and serve as a novel preclinical tool that can achieve greater similarity with human cancer genetics, tumor heterogeneity, and the microenvironment. The PDX cultivation period generally lasts from 14 to 21 weeks. However, most pancreatic cancer patients are already in an advanced stage at the time of diagnosis and have a short survival period. Additionally, the low success rate of PDX cultivation limits its application in pancreatic cancer patients [4].

A recent significant breakthrough in basic cancer research has been the establishment of in vitro 3D tumor cell culture technology. In this process, tumor tissue is directly obtained from patients and rapidly expands in vitro to form patient-derived organoids (PDOs) that are highly similar to the original tissue in terms of physiological and pathological status. PDO is a multi-cellular 3D micro-organ structure that is cultured in vitro on the basis of organoid construction, using human-derived embryonic stem cells, adult stem cells or induced stem cells. Using 3D cell culture technology and the interaction between cells and the matrix, in a culture system with added growth factors, stem cells undergo cell division and spatially restricted differentiation to achieve self-organization in a manner similar to that in the body, forming microorganisms similar to the original tissues or organs of the human body. Organoids have the ability to self-renew and self-organize, as well as to retain and reproduce the genetic heterogeneity, tissue structure, cellular composition, and molecular characteristics of the original tumor; they also have the advantages of a high modelling success rate and short culture time. Today, organoids can be passaged indefinitely and stored in liquid nitrogen for future use and, most importantly, they are genetically stable [5].

The successful construction of pancreatic cancer organoids has provided new avenues and tools for multiple research fields. In the study of disease mechanisms, these organoids simulate the microenvironment and biological characteristics of real tumors, aiding in the in-depth exploration of the occurrence, development, and metastasis mechanisms of pancreatic cancer. In drug screening and personalized medicine, the use of pancreatic cancer organoids for high-throughput drug screening enables the rapid assessment of the cytotoxic effects of different drugs on tumor cells, the discovery of potential therapeutic targets and drug combinations, and the formulation of personalized treatment plans based on patient-specific models. Additionally, pancreatic cancer organoids are easily amenable to genetic editing operations, such as clustered regularly interspaced short palindromic repeats/CRISPR-associated protein 9 (CRISPR/Cas9) technology, which can be used to study the role of specific genes in pancreatic cancer. Finally, pancreatic organoids have potential application value in regenerative medicine and tissue engineering. By optimizing culture conditions and differentiation induction strategies, these organoids can be transformed into functional pancreatic tissue or cells, providing new solutions for the repair and regeneration of damaged pancreatic tissue.

However, the construction of pancreatic cancer organoids still faces many challenges. For example, the creation of organoids requires high-quality tumor samples, which are often difficult to obtain. In addition, various factors, such as the type of biopsy needle used, the number of punctures, and the pathological grade of the patient’s disease, impact the success rate of constructing pancreatic cancer organoids. This article provides a review of the progress in pancreatic cancer organoid research, focusing on the technical aspects of sample collection using endoscopic ultrasound-guided fine-needle aspiration/biopsy (EUS-FNA/FNB) and other influencing factors.

## 2. Development History of Pancreatic Cancer Organoids

**Figure 1:**
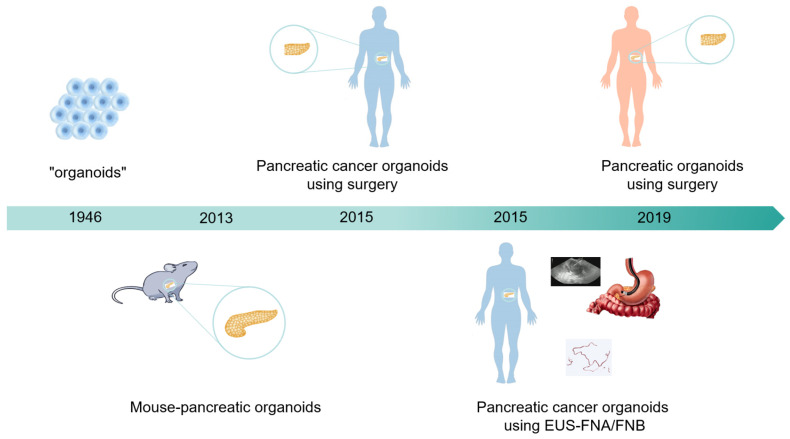
Development history of pancreatic cancer organoids. In 1946, organoids first emerged. In 2013, scientists successfully constructed pancreatic organoids in mice. In 2015, research teams succeeded in constructing pancreatic cancer organoids derived from human surgical and EUS-FNA/FNB sources. In 2019, scientists successfully constructed pancreatic organoids from healthy human bodies, providing a brand-new tool for medical research.

The term “organoids” was first used in 1946 [6]. In 2009, Toshiro et al. established intestinal organoids, and the related results in this field have grown exponentially annually since then [7]. In 2013, Huch et al. constructed pancreatic organoids by isolating ducts from the mouse pancreas [8]. Initially, the isolated ducts were mixed with Matrigel, seeded, and cultured. The culture medium used was AdDMEM/F12 supplemented with B27, N-acetylcysteine, gastrin, and the growth factors EGF, R-spondin 1, Noggin, FGF10, and nicotinamide. Without EGF, R-spondin 1, or FGF10, the cultures deteriorated after 2–5 weeks. Noggin and nicotinamide were essential for maintaining the cultures beyond 2 months (approximately passage 8). Moreover, within one week of seeding and culturing the primary isolated ducts in Matrigel, these organoids can be passaged. These organoids are capable of expansion and passaging for at least 9 months. In 2015, Boj et al. successfully established the first tumor organoids from human PDAC surgical resection samples by optimizing a system in which growth factors, such as R-spondin, were added to promote activation of the WNT signal transduction pathway and induce organoid growth [9]. Organoid formation has a success rate of up to 85%, and organisms can be passaged indefinitely and cryopreserved for future use. These organoids exhibit a complex morphology with varying degrees of dysplastic tall columnar cells, resembling low-grade pancreatic intraepithelial neoplasia (PanIN). In 2019, Romero-Calvo et al. constructed normal human pancreatic duct organoids and precancerous epithelial cell organoids from resected pancreatic specimens. This model was enriched in human-derived biological macromolecules and was used to analyze pancreatic cancer precancerous lesions in rare tissue samples, as well as molecular characteristics and heterogeneity [10] (Figure 1).

Human PDAC organoid samples are collected from surgically resected tumors or through fine-needle aspiration (FNA)/fine-needle biopsy (FNB) [11]. Samples obtained through surgery are larger in volume, have complete cell layer structures, are genetically stable, and exhibit stable drug responses. However, the vast majority of PDAC patients are not candidates for surgery, with only 10% to 20% of pancreatic cancer patients being suitable for surgical intervention, which severely limits the ability to use organoids [12]. Furthermore, neoadjuvant therapy causes PDACs to shrink and disappear [13,14,15]. Therefore, establishing PDAC organoids requires sources other than surgical resection samples. EUS-FNA/FNB can accurately obtain tissue samples from lesions in the pancreas. With real-time ultrasound guidance, this technique can precisely puncture the suspicious pancreatic lesions with a fine needle, ensuring that the obtained samples are highly targeted and representative of the true characteristics of pancreatic cancer tissues. Samples obtained by EUS-FNA/FNB are smaller in volume, have relatively simple cell structures, are prone to genetic variations, and exhibit complex and diverse drug responses (Table 1). In clinical practice, EUS-FNA has become the preferred method for diagnosing pancreatic cancer, especially in patients with locally advanced unresectable and metastatic pancreatic cancer [16]. EUS—FNA/FNB samples are often preferred due to their minimal invasiveness, accessibility, repeatability, low risk of complications, and high cost-effectiveness. Compared with surgical samples, they exhibit significant advantages in preserving tissue architecture, genetic stability, reducing the risk of contamination, and enhancing patient comfort. Compared to surgical samples, pancreatic cancer organoids constructed from EUS-FNA samples have a higher growth efficiency [17]. The first pancreatic cancer organoid model was successfully established from a small amount of tissue obtained via endoscopic biopsy by optimizing the modelling conditions. This model promoted the construction of organoids derived from tissue from non-surgical pancreatic cancer patients [9].

## 3. Key Technical Steps for Successfully Establishing Pancreatic Cancer Organoids via EUS-FNA/FNB

### 3.1. Pancreatic Cancer Puncture

Patients suspected of having pancreatic cancer undergo an EUS examination, and an appropriate needle is selected for puncture; an FNB needle is the primary choice because it can obtain more tissue. Concurrently, rapid on-site evaluation (ROSE) of cytopathology is performed. Once a preliminary positive diagnosis is obtained and an adequate number of cells are collected, an additional one to two punctures via EUS-FNB are performed to collect tissue for organoid creation. The samples are also subjected to pathological, immunohistochemical, and cytological testing.

### 3.2. Establishing Pancreatic Cancer Organoids

After tissue samples are obtained through ultrasound-guided puncture, they are placed in basal medium (Advanced DMEM/F-12 supplemented with 1% penicillin/streptomycin, 1% GlutaMax, and 10 mM HEPES) and transported on ice. The red blood cells and fat are removed from the tissue samples using cold wash medium, and the samples are centrifuged at 400× *g* for 5 min. The digestion solution (collagenase and dispase II dissolved in sterile wash medium to a concentration of 0.125 mg/mL each, supplemented with DNase I) is pre-warmed to 37 °C and added to the tissue samples, and the mixture is incubated on a shaker at 37 °C for 15–30 min. Digestion is terminated by adding three times the volume of ice-cold washing medium to the digesting solution.

The mixture is subsequently centrifuged at 400× *g* for 5 min at 4 °C. An appropriate amount of red blood cell lysis buffer is added to the pellet, and after 3–5 min, the lysis reaction is terminated with cold wash medium. The mixture is centrifuged again under the same conditions, and the supernatant is discarded. The pellet is placed on ice, gently mixed with pre-chilled Matrigel using a pre-cooled pipette tip, and then quickly seeded onto a 24-well plate. Once the Matrigel solidifies, organoid initiation medium (OIM) is added, and the mixture is cultured in a 37 °C CO_2_ incubator. On the third day of culture, the medium is replaced with organoid growth medium (OGM). Pancreatic cancer organoids are passaged, cryopreserved, and revived. The successful establishment of organoid cultures depends on the ability of the organoids to be passaged to at least the fifth generation.

The preparation of OIM and OGM is outlined in Table 2.

### 3.3. Identifying Pancreatic Cancer Organoids

**Figure 2:**
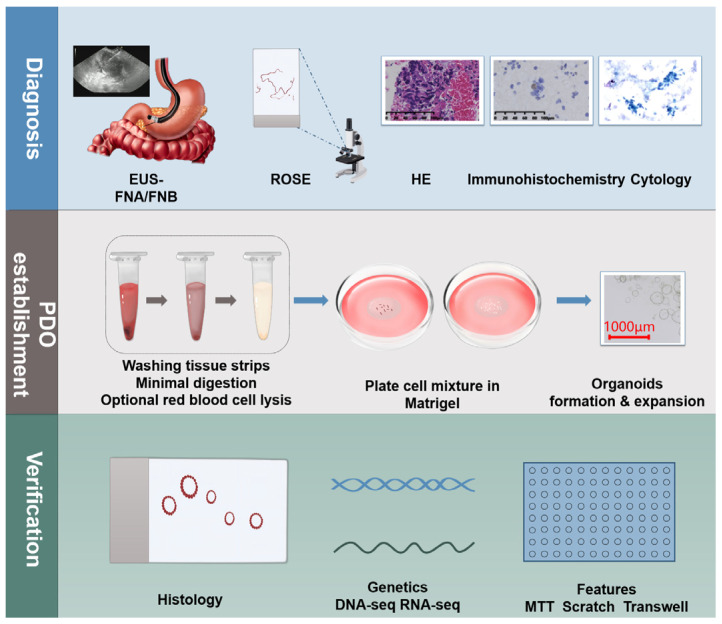
Establishing pancreatic cancer organoids using EUS-FNA/FNB. Suspected pancreatic cancer patients undergo EUS-FNB puncture to obtain tissue samples, which are then subjected to ROSE and various tests, including pathology, immunohistochemistry, and cytology. Sample processing involves washing, digestion, centrifugation, and lysis, followed by mixing with matrix gel for inoculation and culture, ultimately forming pancreatic cancer organoids. These organoids must be passaged at least to the fifth generation and undergo pathological, genetic, and functional characterization to ensure similarity to primary pancreatic cancer.

After the successful establishment of pancreatic cancer organoids, a series of identifications and comparisons are necessary to ensure that their biological characteristics are similar to those of the primary pancreatic cancer in human patients. These identifications include the observation of pathological features, the analysis of genetic characteristics, and the assessment of functional properties. In terms of pathology, the tissue structure and specific protein expression of the organoids are observed through hematoxylin and eosin (HE) and immunohistochemical staining. In terms of genetics, next-generation sequencing technology is used to detect the gene mutation spectrum of the organoids and compare it with that of primary pancreatic cancer samples. In terms of functional properties, methods, such as 3-(4,5-dimethylthiazol-2-yl)-2,5-diphenyltetrazolium bromide (MTT) assays, scratch tests, and Transwell invasion assays, are used to evaluate the proliferative, migratory, and invasive abilities of the organoid cells, respectively. Through these multifaceted identifications and comparisons, a comprehensive assessment of the accuracy and reliability of the pancreatic cancer organoid model can be performed, providing a solid foundation for subsequent basic research and clinical translation (Figure 2).

EUS-FNA/FNB has shown great potential in the construction of pancreatic cancer organoids, providing new avenues for the research and treatment of pancreatic cancer. However, despite certain successes achieved with this technology, many factors can affect the success rate of organoid construction in practical operations.

## 4. Factors Influencing the Success of PDAC Organoid Construction

**Figure 3:**
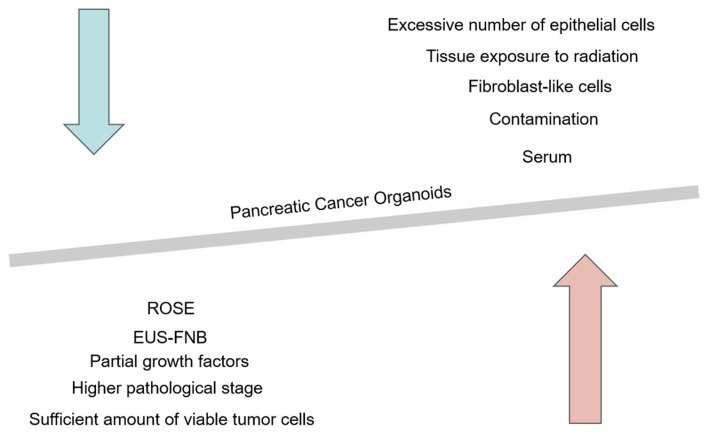
Possible factors affecting the success rate of constructing pancreatic cancer organoids. The red arrow represents the key factors that may improve the success rate of constructing pancreatic cancer organoids, including the use of FNB needles for puncture to collect more tissue samples, ensuring a sufficient number of viable cells, as well as selecting patients with higher pathological grades, culture media rich in growth factors, and patients with positive ROSE results. The blue arrow represents the unfavorable factors that may reduce the success rate of constructing pancreatic cancer organoids, such as the choice of serum components in the culture medium, potential contamination during the puncture process, interference from fibroblast-like cells, an excess of epithelial-like cells, and tissue exposure to radiation.

In recent years, with the support of EUS-FNA/FNB technology, researchers have successfully constructed multiple pancreatic cancer organoids, as shown in Table 3 below. The definition of successful PDO establishment varies among studies, making direct comparisons of PDO generation efficiency across different studies complex. The appearance of organoid structures within the first three generations or successful proliferation after the fifth generation both indicate successful organoid construction [17,18]. The establishment rate of PDOs from primary tissue ranges from 40–70%, and further exploration of factors that may affect the success rate is needed [17,19]. High-quality sample sources, sterile operations, and optimized culture medium formulations (containing specific growth factors and small molecules) are key factors. Individual patient differences and disease stages may also affect the formation of organoids. By considering these factors comprehensively, the success rate of PDAC organoid construction can be improved, providing more reliable models for research and treatment (Figure 3).

### 4.1. Impact of Sample Quality on the Successful Construction of Pancreatic Cancer Organoids

Tumor specimen size, cellularity, and profibrotic status also significantly influence the establishment of PDOs [20,29]. Hogenson et al. reported that the success rate of constructing pancreatic cancer organoids was 13% (2/16). The main reasons for the failure of organoid formation were the low number of tumor cells and the fact that fibroblast-like cells seemed to grow excessively and inhibited organoid growth. A standardized process for measuring specimen tumor cellularity during collection will increase the success rate of PDO establishment [26]. In this study, the successful establishment of PDOs depended on the number of viable cells obtained after digestion, with at least 100,000 viable cells being necessary for the establishment of PDOs in PDAC [33]. The choice of digestive enzymes and the duration of digestion also have a significant impact. Proper selection of digestive enzymes and strict control of digestion time can ensure that the tissue is effectively digested into single cells or cell clusters without compromising cell viability. This is crucial for the subsequent formation of organoids.

Although third-generation EUS-guided fine needles have recently demonstrated higher histological core collection rates and higher genetic testing success rates, sample sizes remain limited [34]. To obtain sufficient tissue, multiple punctures are performed using thicker needles, which can lead to serious complications, such as bleeding [35,36], suggesting that the number of punctures should be reduced. Ikezawa et al. successfully constructed PDAC organoids using residual samples from saline flushing (RSSF) during EUS-FNA, with a success rate of 80% (4/5). However, the study sample size was small, and it is unclear whether EUS-FNA RSSF significantly reduces the number of punctures required to create PDAC organoids [27]. In this study, a single puncture with a 22G EUS-FNB needle was sufficient to establish PDOs [33].

Differences in pancreatic cancer organoid success rates may be due to differences in the procedure for obtaining tissue samples [20], including the size and type of the needle, the use of probes and suction, the application of the fanning technique, the size and location of the tumor, the experience of the endosonographer, and the choice to use an aspiration syringe to obtain samples or to employ the slow-pull technique. These factors may cause cell damage and contamination of blood or epithelial cells [37]. Lacomb et al. reported that the failure to establish pancreatic cancer organoids from EUS-FNA specimens may be due to the excessive number of epithelial cells, which prevents the proliferation of the organoids [18]. In a multicenter collaboration, Seppälä reported that 78% (35/45) of samples successfully generated organoids. However, there is no way to transition from the expansion stage to the characterization stage, and expansion failure may be due to “contamination” by the growth of biomass derived from normal ductal epithelium or microbial contamination [17].

Although EUS-FNA/FNB technology has advanced, challenges related to tissue acquisition, such as insufficient cells and low viability, still affect the success rate and reproducibility of PDO. To address these issues, it is necessary to optimize biopsy techniques, enhance the proficiency of operators and use advanced imaging guidance, improve sample processing methods, strengthen quality control, establish cell quality assessment standards and monitor the PDO construction process, and develop specific protocols based on patients’ tumor profiles and optimize culture conditions. In summary, in terms of sample quality, the source, freshness, and viability of pancreatic cancer tissue are crucial for the establishment of organoids. Samples that are well-sourced, fresh, and have high tumor cell viability are more conducive to organoid culture. When EUS-FNA/FNB is used to collect samples from pancreatic cancer patients, tissues with a sufficient viable cell volume should be obtained with as few punctures as possible, and the tissues should be kept viable and sterile during the transfer process to improve the success rate of pancreatic cancer organoid formation.

### 4.2. Impact of Medium and Additive Selection on the Successful Construction of Pancreatic Cancer Organoids

Several studies have reported on the use of samples obtained by EUS-FNB to establish pancreatic organoids [20,38,39]. Some of these studies have shown that PDAC is significantly heterogeneous among patients [38,40]. This heterogeneity occurred because the PDAC organoid media used in each study varied and contained multiple growth factors or inhibitors that allowed the growth of not only cancer cells with specific genetic mutations but also normal cells [41]. Ishida et al. standardized the pancreatic cancer patients for PDAC organoid culture using specimens obtained via EUS-FNB and achieved a success rate of 63.2% for a PDAC organoid culture [21]. Personalizing organoid media (e.g., EGF-depleted media enriched with KRAS mutants) may increase PDO formation and eliminate non-tumor “contaminants” that may inhibit PDO growth [38]. The success rate of PDO generation in Ikezawa’s study was 80%, and in these patients, organoids were also established with the KRAS-selection medium. However, the sample size of this study was only five people [27]. The enrichment of markers associated with PDO dedifferentiation in WNT medium may alter PDO phenotypes [42]. Furthermore, compared with PDOs grown in preparation appropriate for tumor organoid medium (PaTOM) [24], PDOs grown in WNT medium are generally more resistant to chemotherapy and targeted therapies and are enriched in E2F targets and the epithelial–mesenchymal transition (EMT) and TGF-β pathways, which are associated with increased drug resistance [26]. Adding serum to the culture medium is not conducive to PDO isolation and propagation efficiency [38]. Therefore, the culture conditions are also one of the key factors. Environmental parameters, such as the composition of the culture medium, temperature, oxygen concentration, and nutrient supply need to be precisely controlled. Pancreatic cancer organoids of different subtypes may have different requirements for culture conditions, and we need to continuously optimize these conditions to adapt to different situations. When constructing pancreatic cancer organoids, the selection of appropriate culture media and additives is one of the key factors for improving the success rate.

### 4.3. Effects of FNA vs. FNB on the Successful Construction of Pancreatic Cancer Organoids

FNA typically uses thinner needles, which are suitable for obtaining cellular samples but may result in insufficient cell numbers and cell damage, potentially reducing the success rate of organoid construction. In contrast, FNB uses thicker needles that can obtain larger tissue samples, increasing the number of viable cells and aiding in the successful construction of organoids. Therefore, choosing the appropriate needle type is crucial for optimizing the establishment of pancreatic cancer organoids. Demyan et al. reported that the samples of 29 patients diagnosed with PDAC by histopathological evaluation were obtained through FNB, while those of 14 patients were obtained through FNA. The success rates of PDO construction were 56% and 53%, respectively. The two needle types used for FNA and FNB were both effective, and there was no significant difference in the success rate of pancreatic cancer organoid construction (*p* = 0.215) [24]. A total of 17 samples (34%) in the study by Wiessner et al. resulted in successful PDO construction. Among them, nine samples were obtained by FNB, two samples were obtained by FNA, and six samples were obtained by both FNA and FNB. Thirteen of the seventeen PDOs (76%) were malignant. EUS-FNB samples were superior to EUS-FNA samples in successfully generating PDOs, although the difference was not statistically significant [30].

### 4.4. Influence of Pathological Grade on the Success Rate of Pancreatic Cancer Organoid Construction

Organoids can be constructed through EUS-FNA sampling, which can theoretically be used in pancreatic cancer patients regardless of their stage [43]. Several studies reported that the success rate of PDO generation was independent of tumor stage and size [27,33]. However, in one study, biopsy samples collected from 4 patients prior to neoadjuvant therapy (NAT) were sent for PDO development, with a success rate of 75%. All 3 patients with successful PDOs had stage III (American Joint Committee on Cancer [AJCC] 8th edition) disease and had borderline-resectable pancreatic cancer (BRPC) at surgical staging [24]. In the study by Lee et al. [28], only 1 of the 12 (12/20, 60%) patients with successfully constructed PDOs had not received first-line chemotherapy (TNM stage, III/IV [5/7]). In the study by Kim et al. [31], the pathological stages of the patients with successful PDOs (94/113, 83.2%) were I: 14; II: 7; III: 33; and IV: 59. High-grade tumors typically have greater cell proliferation and greater invasiveness, which may make these tumor samples more likely to form organoids when cultured in vitro. Therefore, pathological grade may affect the success of pancreatic cancer organoid construction [31].

### 4.5. Impact of ROSE on the Success Rate of Pancreatic Cancer Organoid Construction

ROSE is a method of evaluating tissue samples in real time during surgery, helping surgeons and pathologists quickly judge the quality and suitability of the sample collected. Tiriac et al. first used ROSE with tissue samples obtained by FNA puncture to make a positive preliminary diagnosis and obtain a sufficient number of cells. After the FNB needle was inserted into the mass, the “slow pull” technique of the inner needle was used to perform several acupunctures; 1 to 2 punctures were used to create organoids, with a success rate of 25/38 (66%) [20]. Ishida et al. used excess samples and very little culture medium, which led to sampling and technical errors that had a substantial impact, but the success rate of organoid culture was still 24/38 (63.2%). PDAC organoids can be cultured even in patients with pathologically inconclusive EUS-FNB results, which facilitates the clinical diagnosis of PDAC [21].

In addition, tissue exposure to radiation is the factor most closely related to the failure of organoid establishment. However, the organoid samples in that study were derived from surgically resected tissue. Radiology researchers have successfully constructed pancreatic cancer organoids from ultrasound-guided puncture samples. The impact has not yet been elucidated. Establishing PDOs from chemotherapy-naïve and post-NAT tissues allows longitudinal generation of PDOs at different times during disease progression in the same patient. These matched PDOs are valuable models for explaining tumor evolution and changes in drug response [24].

## 5. Applications of Pancreatic Cancer Organoids

### 5.1. Application of Pancreatic Cancer Organoids in the Study of Disease Occurrence and Development Mechanisms

Boj et al. first reported 3D organoid models of mouse and human pancreatic cancer. Orthotopic transplantation into mice resulted in invasive cancers composed of ill-defined and invasive glands, whereas PanIN-like structures and PDAC clearly induced a desmoplastic response. Orthotopic transplantation of the organoids resulted in normal disease progression and a mesenchymal cell response with low blood vessel density typical of pancreatic cancer, whereas 2D tumor cells typically do not exhibit this mesenchymal cell response when transplanted in vivo. These characteristics indicate that human pancreatic cancer organoids can better reflect the occurrence and development process of pancreatic cancer [9]. Yea Ji et al. performed organoid studies and transcriptome analysis with a large-scale human PDAC organoid cohort to determine the molecular mechanisms that promote invasion. The results of that study revealed the changes in molecules and cells that promote the invasion of human pancreatic cancer, providing a theoretical basis and potential therapeutic targets for inhibiting the invasion of pancreatic cancer. The above studies all show that pancreatic cancer organoids provide a new platform for research on the underlying mechanisms of the occurrence and development of pancreatic cancer [44].

### 5.2. Application of Pancreatic Cancer Organoids in Drug Screening and the Study of Drug Resistance Mechanisms

Duan et al. used pancreatic cancer organoids to conduct drug screening and successfully identified a drug called perhexiline maleate from more than 6,000 compounds that can potently inhibit the growth of pancreatic cancer organoids [45]. Takeuchi et al. used mesenchymal stem cells derived from human induced pluripotent stem cells and fused patient-derived PDAC cells to generate a new pancreatic cancer organoid. This innovative fusion method produced two different pancreatic cancer organoids, which simulated the patient’s tumor tissue and unique tumor microenvironment well and may aid in screening anticancer drugs [46]. In addition, Sandhya et al. used patient-derived pancreatic cancer organoid models to establish a precision cancer medical platform to evaluate the sensitivity of tumors to different drugs before and after standard chemotherapy to improve treatment effects in clinical settings [47].

Another researcher cocultured PDAC organoids with cancer-associated fibroblasts and reported that the proliferation of PDAC organoids was increased and that chemotherapy-induced cell death was reduced, suggesting that the pancreatic cancer PDO model can be used not only to analyze the drug response spectrum but also to elucidate the molecular mechanisms by which drug resistance supports the role of the tumor matrix [48]. Farshadi et al. constructed human PDAC organoid models from patients who had not received treatment and those who had received 8 weeks of FOLFIRINOX treatment and exhibited drug resistance. The results revealed that the molecular mutations and histopathological characteristics of the organoids after FOLFIRINOX treatment were different from those of the original tissue. The organoids also exhibited a high degree of consistency in terms of drug resistance, and the drug resistance was also consistent with that observed in clinical practice [49].

### 5.3. Role of Pancreatic Cancer Organoids in Personalized Medicine and Gene Editing Research

Personalized therapy is a trend in tumor treatment, and pancreatic cancer organoid models can be used to conduct targeted research and screen targeted drugs. In terms of drug development, exposing organoids to different chemotherapeutic agents can accurately predict the responses of various patients to these drugs. This helps doctors tailor the most effective chemotherapy regimen for each patient, avoiding the side effects caused by the use of ineffective drugs and thereby enhancing the precision of treatment [22,23]. In disease modelling and mechanism research, these organoids retain the genetic characteristics, cellular heterogeneity, and microenvironmental features of the patient’s tumor. This allows researchers to delve deeply into the mechanisms of pancreatic cancer development, identify new therapeutic targets, and provide a basis for developing unique treatment strategies tailored to individual patients [17,24]. In addition, patient-derived organoids can also be used to evaluate the effectiveness of treatments, such as surgery. For example, by conducting simulated treatments on organoids before surgery and observing their impact on tumors, doctors can better predict the feasibility and effectiveness of the surgery in advance. This allows for the optimization of surgical plans and the realization of truly personalized surgical treatments [19,26,29].

By using CRISPR-Cas9 genome editing and drug screening to characterize the genetic interactions of drugs with ARID1A and BRCA2, a study revealed that missense mutations in the PDAC driver gene ARID1A were associated with increased sensitivity to the kinase inhibitors dasatinib and VE-821 [50].

### 5.4. Application of Pancreatic Organoids in Regenerative Medicine and Tissue Engineering

When human pancreatic organoids were xenografted into the pancreas of immunodeficient mice, the organoids survived for a long time in the mouse body and showed no signs of tumor development [51]. When human pluripotent stem cell-derived acinar/ductal organoids were transplanted into immunodeficient mice, the organoids developed normal pancreatic ductal and acinar tissue similar to that of the human fetal pancreas, with no evidence of tumor formation or transformation [52]. Pancreatic organoids play important roles in developing regenerative medicine therapies for diabetes and disease modelling.

Researchers used organoid technology combined with bioscaffolds that mimic perfusable vascularized blood vessels. When gemcitabine was perfused via blood vessels into the pancreatic tumor stroma, the drug was less effective than when it was applied directly to tumor organoids [53]. Combined with these bioscaffolds, pancreatic organoids can also be used to study the mechanism of drug action in the tumor microenvironment, providing new ideas for the development of more effective anticancer drugs.

## 6. Development Prospects of Pancreatic Cancer Organoids

**Figure 4:**
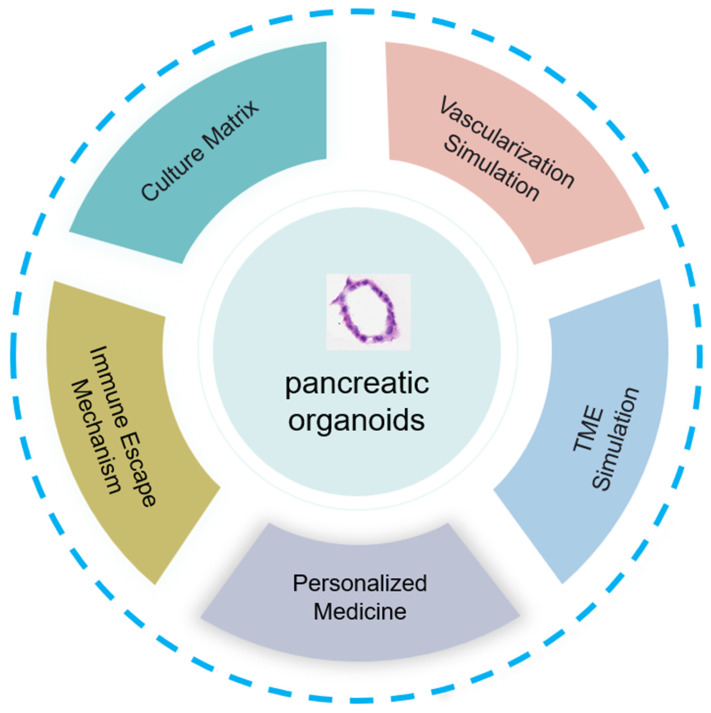
The development of pancreatic organoids involves key aspects, such as innovation in culture matrix materials, vascularization mimicry, tumor microenvironment simulation, research on immune evasion, and application in personalized medicine, demonstrating their full potential and impact.

Organoids are most commonly established in Matrigel, but the composition of Matrigel is not fully defined, which may affect subsequent research. Tumor organoid culture systems often use animal-derived Matrigel, which contains unknown extracellular components that may alter cell and organoid behavior. Well-defined peptide hydrogel materials may solve this problem. The peptide hydrogel material CulX II closely simulates the natural extracellular matrix and is suitable for the construction and culture of tumor organoids. However, the tumor organoids established by Huibin Wang and others are currently immature, and the cell composition and structure of the tumor organoids are still different from those of real tumors. In the future, new self-assembled peptide hydrogel materials should be developed, in vitro culture conditions should be optimized, a more complete tumor organoid culture system should be established, and tumor organoids that are more consistent with real tumor tissues in terms of cell composition, structure, and phenotype should be obtained (Figure 4).

The vascularization of organoids is another aspect to consider. To overcome the lack of vascular circulation in organoids, researchers have cocultured organoids with endothelial cells in a microfluidic device [54]. Developing “organoid-on-a-chip” technology by combining human organoids with organ-on-a-chip engineering enables different types of organoids to be cultured individually and allows them to communicate for preclinical drug screening [55].

Organoid models of pancreatic cancer lack interactions with tumor microenvironment components, such as blood vessels, blood, immune cells, and microbiota. Some studies have used cocultures of immune cells or fibroblasts to compensate for this shortcoming [48,56]. A coculture system of organoids and immune cells was established for screening immunosuppressants and evaluating the sensitivity of tumors to immunotherapy drugs [57,58]. In addition, 3D coculture of organoids with stromal cells and tumor-infiltrating lymphocytes is a cutting-edge method that can recapitulate the tumor microenvironment and target drugs related to immune pathways, providing a basis for clinical research [56]. The introduction of the InterOMaX platform has opened up a new perspective for understanding the complex interactions within the tumor microenvironment. This platform leverages a 3D coculture system, enabling researchers to simulate the dynamic interactions between pancreatic cancer cells and the matrix in an environment that closely mimics physiological conditions. With technological advancements, future improvements to the InterOMaX model system will focus on enhancing its simulation accuracy and functional diversity [59].

Organoids are also used to determine the mechanisms by which tumors evade immune system attack, which reveals new immune escape pathways and provides clues for the development of novel immunotherapy strategies. Additionally, this model is suitable for testing the effects of immunotherapy in combination with other treatment methods (such as chemotherapy and radiotherapy) to find the best combination. In the early stages of drug development, the use of organoids for toxicity testing not only reduces the need for animal experiments but also increases the speed and accuracy of drug safety assessment. Furthermore, organoid technology enables samples obtained from patients to be cultured into three-dimensional tumor structures in vitro, which is crucial for achieving true personalized medicine. Physicians can tailor treatment plans on the basis of the organoid response to specific immunotherapies, thereby optimizing therapeutic outcomes and reducing unnecessary side effects.

For diseases that are rare or for which it is difficult to obtain sufficient clinical samples, organoids provide a valuable disease modelling system that helps scientists to better understand the biological characteristics of the disease and potential therapeutic targets. By analyzing the differences in the responses of organoids to various treatments, it is also possible to discover biomarkers that predict responses to immunotherapy. Furthermore, organoids are used to explore the mechanisms by which tumors develop resistance to immunotherapy, which is highly important for overcoming treatment resistance. Finally, as an educational tool, organoids enable medical students and physicians to visually learn about complex biological processes and disease mechanisms, promoting advancements in medical education and clinical practice. With the continuous development and refinement of technology, it is anticipated that organoids will play an increasingly pivotal role in precision medicine and personalized treatment in the future.

In personalized medicine, it is crucial for PDOs to accurately reflect individual tumor heterogeneity. However, there are currently issues with validation processes and consistency across different laboratories. For example, sample collection methods have their respective strengths and weaknesses, culture conditions vary significantly, and detection techniques (including inconsistent morphological, molecular biomarker, and functional testing standards) differ. These factors lead to poor comparability of research results and uncertainty in clinical applications. There is an urgent need to establish unified standards and guidelines, enhance collaboration and communication, build collaborative networks, conduct multicenter studies, and organize conferences and training workshops. Strengthening regulatory oversight and quality control is also essential to address these issues and promote the application of PDOs in personalized medicine.

In the research field of pancreatic cancer organoids, currently, information about their long-term genetic stability and representativeness of disease progression compared to fresh tumor samples is quite limited. Although some preliminary signs have been observed that the key gene expression and cell characteristics of organoids do not change significantly over a period of time, it is still necessary to deeply explore the mechanism of genetic stability maintenance. In the future, advanced gene sequencing technologies can be applied to conduct genetic analyses of organoids at different time points to monitor changes in genetic material. At the same time, more qualified samples should be obtained for a detailed multi-angle comparison with fresh tumor samples in terms of gene expression, cell differentiation, histopathology, etc., in order to clarify the advantages and disadvantages of organoids in simulating disease progression, support pancreatic cancer research, and bring new hope to patients.

## Figures and Tables

**Table 1:** Comparison of morphological, genetic stability, and drug response differences between organoids from different sources.

Source of Organoids		Surgical Resection	EUS-FNA/FNB
Morphological differences	Size and shape	Larger in size, more regular in shape, closer to the structure of the primary tissue	Smaller in size, varied in shape due to tissue fragmentation during the puncture process
	Cell layers and structure	More complete cell layers and structure, favorable for maintaining the original tissue architecture	Relatively simpler in cell layers and structure, but still retain certain features of pancreatic cancer tissue
Genetic stability differences	Gene expression and mutations	Better retention of the gene expression patterns and mutation characteristics of the primary tissue, higher genetic stability	Possible changes in gene expression patterns and mutation characteristics due to mechanical stress or chemical factors during the procedure
	Genetic variation and evolution	Slower genetic variation and evolution speed	May undergo more passages and expansions, leading to higher genetic variation and evolution speed
Drug response differences	Sensitivity and resistance	May have higher sensitivity or resistance to certain anticancer drugs	Different response to drugs, showing unique sensitivity or resistance characteristics
	Reaction dynamics	More stable reaction dynamics, better preservation of tissue structure and cell–cell interactions	More complex and variable reaction dynamics, possible disruption or alteration of cell–cell interactions and tissue structure

**Table 2:** Preparation of OIM and OGM.

Component Name	Pan1creaCult™ OIM + PGE2 + Y-27632	PancreaCult™ OGM + PGE2
PancreaCult™ organoid basal medium (human)	84.65%	94.75%
PancreaCult™ organoid growth supplement (human)	5%	5%
Organoid supplement	10%	——
EGF (100 µg/mL)	0.05%	0.05%
Gentamicin (optional) (50 mg/mL)	1%	0.1%
PGE2 (1 mM) (immediately before use)	1%	0.1%
Y-27632 (10 mM) (immediately before use)	1%	——

**Table 3:** Establishing pancreatic cancer organoids from EUS-FNA/FNB biopsy specimens.

Number	Needle Type	Number of Needle Passes	Location	Number of Examples	SR [%]	Pathological Grade	Digestion Solution	Medium	Organoid Culture Time
Tiriac [20]	22G FNB	1–2 times	Head: 23; body/tail: 15	38	66%	Unspecified	5 mg/mL collagenase ×I, 10 μg/mL DNAse I, 10.5 μM Y-27632 in human complete feeding medium	Advanced DMEM/F12, HEPES 10 mM, GlutaMax 1×, A83-01 500 nM, hEGF 50 ng/mL, m-Noggin 100 ng/mL, hFGF10 100 ng/mL, hGastrin I 0.01 μM, N-acetylcysteine 1.25 mM, Nicotinamide 10 mM, PGE2 1 μM, B27 supplement 1× final, R-spondin1-conditioned media 10% final, and afamin–Wnt3A-conditioned media 50% final	≥5 passages of growth (P5)
Ishida [21]	22G/25G FNB	Unspecified	Unspecified	38	63.2%	Unspecified	Indigestion	DMEM/Hams F12/MCDB105 (2:1:1 ratio), 12% FBS (Gibco, USA), and B27-supplement (Gibco)	Not reported
Armstrong [22]	Unspecified	Unspecified	Unspecified	18	83%	Unspecified	1:3 ratio of Advanced DMEM/F12 and TrypLE Express, supplemented with collagenase ×I 0.012% (*w*/*v*), 0.012% dispase (*w*/*v*), 10.5 µM Y-27632, and 10 mg/mL DNAse I	Advanced DMEM/F12, 1 M HEPES, 1× B27-supplement, 1× N2-supplement, 1 mM N-Acetylcysteine, 10 mM nicotinamide, hGastrin 0.1 µmol/L, hEGF 50 ng/mL, 500 nM A83-01, Y-27632, 100 ng/mL hFGF-10, and Wnt3A–R-spondin1–Noggin-conditioned media (50% of final volume)	Not reported
Beutel [23]	19G FNB	Unspecified	Unspecified	2	100%	Resectable: 1; metastasized: 1	Accutase	Organoid culture medium containing WNT3A/RSPOI-conditioned medium	Not reported
Demyan [24]	14G FNA	1 time	Unspecified	14	53%	Unspecified	1 mg/mL collagenase ×I, 10 μg/mL DNAse I, 10.5 μM Y-27632 in human complete medium	Human complete feeding medium: advanced DMEM/F12, HEPES 10 mM, GlutaMax 1×, Primocin 1×, A83–01 500 nM, hEGF 50 ng/mL, m-Noggin 100 ng/mL, hFGF10 100 ng/mL, hGastrin I 0.01 μM, N- acetylcysteine 1.25 mM, nicotinamide 10 mM, B27 supplement 1× final, R-spondin1-conditioned media 10% final, and afamin–Wnt3A-conditioned media 50% final	Not reported
Demyan [24]	29G FNB	1 time	Unspecified	29	56%	Unspecified	1 mg/mL collagenase ×I, 10 μg/mL DNAse I, 10.5 μM Y-27632 in human complete medium	Human Complete Feeding Medium: advanced DMEM/F12, HEPES 10 mM, GlutaMax 1×, Primocin 1×, A83–01 500 nM, hEGF 50 ng/mL, m-Noggin 100 ng/mL, hFGF10 100 ng/mL, hGastrin I 0.01 μM, N-acetylcysteine 1.25 mM, Nicotinamide 10 mM, B27 supplement 1× final, R-spondin1-conditioned media 10% final, afamin–Wnt3A-conditioned media 50% final	Not reported
Grossman [19]	22G FNB	Unspecified	Unspecified	28	11%	Unspecified	Digested with STEMxyme-1 (250 CLS units per mL) for 30 to 40 min. Pellets were further digested with Accutase for 30 min.	Growth medium containing Y-27632, 5% Matrigel, and growth factors, such as insulin and FGF2.	≥2 passages of growth (P2)
Hennig [25]	FNA	Unspecified	Unspecified	6	83%	Unspecified	Digested with dispase II (2.5 mg/mL, Roche, Basel, Switzerland) and collagenase II (0.625 mg/mL, Sigma-Aldrich, Germany) in DMEM/F12+++ medium (DMEM/F12 (Invitrogen, USA) supplemented with 1× HEPES (Invitrogen), 1× Pen/Strep (Invitrogen), and 1×GlutaMax (Invitrogen))	Cultivated in human PDAC organoid medium DMEM/F12+++ supplemented with Wnt3a-conditioned medium (50% *v*/*v*), noggin-conditioned medium (10% *v*/*v*), RSPO1-conditioned medium (10% *v*/*v*), B27 (1×, Invitrogen), nicotinamide (10 mM, Sigma-Aldrich), gastrin (1 nM, Sigma-Aldrich), N-acetyl-L-cysteine (1 mM, Sigma-Aldrich), Primocin (1 mg/mL, InvivoGen), recombinant murine epidermal growth factor (mEGF, 50 ng/mL, Invitrogen), recombinant human fibroblast growth factor 10 (hFGF10, 100 ng/mL, PeproTech, USA), A-83-01 (0.5 μM, Tocris Bioscience, UK), and N2 (1×, Invitrogen).	≥10 passages of growth (P10)
Hogenson [26]	FNA	Unspecified	Unspecified	16	13%	Unspecified	Digested using a human Tumor Dissociation Kit (Miltenyi Biotec, Germany, 130-095-929) in a gentleMACS Octo Dissociator with Heaters (Miltenyi Biotec), filtered through a MACS SmartStrainer (100 μm) filter (Miltenyi Biotec, 130-098-463)	PaTOM medium contains DMEM plus GlutaMax (Gibco, 10564-011), 0.1% penicillin–streptomycin–amphotericin B solution, 0.25 μg/mL hydrocortisone (Sigma-Aldrich, H0888), 1% B27 (Gibco, 12587-010), 50 μg/mL L-ascorbic acid (Sigma-Aldrich, A92902), 20 μg/mL insulin (Sigma-Aldrich, I9278), 100 ng/mL FGF2 (R&D Systems, USA, 233-FB), and 100 nM all-trans retinoic acid (Sigma-Aldrich, R2625). PDO culture medium consists of PaTOM media plus 10 μM Y-267632 (ROCK inhibitor) (Selleckchem, USA, S1049) and 5% Matrigel. WNT medium contains 50% L-WRN-conditioned media (Wnt3a, R-spondin, and Noggin) (53), 50% Advanced DMEM/F12 (Gibco, 12634-010), 1× HEPES (Gibco, 15630-080), 1× GlutaMax (Gibco, A12860-01), 1× N2 Supplement (Gibco, 17502048), 1× B27 Supplement (Gibco, 12587-010), 50 ng/mL EGF (R&D Systems, 236EG200), 3 μM SB202190 (Selleckchem, USA, S1077), 500 nM A-83-01 (Selleckchem, S7692), 1 mM N-acetylcysteine (Sigma-Aldrich, A9165), 10 mM nicotinamide (Sigma-Aldrich, N3376), 10 nM gastrin I (Sigma-Aldrich, G9020), 100 ng/mL FGF10 (Peprotech, USA, 100-26), 100 μg/mL Primocin (Invivogen, ANT-PM-1), and 1% penicillin–streptomycin–amphotericin B solution.	≥3 passages of growth (P3)
Ikezawa [27]	22G/25G FNA	2–4times	Head: 3; body/tail: 2	5	80%	I: 1; III: 3; IV: 1	2.5 mg/mL Liberase TH (MERCK, Germany; 5401135001) and 10 μg/mL DNase I (MERCK; DN25)	Advanced DMEM/F12 supplemented with 1× GlutaMax (Thermo Fisher Scientific, USA; 35050061), 10 mM HEPES (Thermo Fisher Scientific; 15630080), 100 U/mL penicillin–streptomycin (Thermo Fisher Scientific; 15140122), 50 μg/mL Primocin (InviivoGen; 14860–94), 1× B27 supplement (Thermo Fisher Scientific; 17504044), 50 ng/mL epidermal growth factor (EGF; Thermo Fisher Scientific; PMG8044), 50 ng/mL fibroblast growth factor-2 (FGF2; PeproTech, USA; 100–18B), 100 ng/mL insulin-like growth factor 1 (IGF1; BioLegend; 590904), 1.25 mM N-acetylcysteine (MERCK; A9165), 10 nM gastrin (MERCK; G9145), 1 μg/mL R-spondin (PeproTech; 120–38), 5 μM A83–01 (TGF-β inhibitor) (Tocris Bioscience, U.K; 2939), 10 μM Y-27632 (FUJIFILM, Japan; 259–00613), 10 % afamin–Wnt3a CM (MBL, Japan; J-ORMW301 R), and 100 ng/mL Noggin (BMP inhibitor) (PeproTech; 250–38).	≥5 passages of growth (P5)
Lee [28]	19G/22G FNA	2–3times	Head: 5; body: 2; tail: 5	20	60%	Unspecified	Indigestion	Organoid culture medium: complete medium: 50% (*v*/*v*) with Wnt-3A, R-spondin1, and m-Noggin-conditioned medium, human epidermal growth factor 50 ng/mL, human fibroblast growth factor-10 100 ng/mL, nicotinamide 10 mM, 500 nM A83-01, 1× B27 supplement, N-acetylcysteine 1.25 mM, 10% fetal bovine serum (FBS), and human gastrin I 0.01 μM in 50% basal culture medium. Conditioned medium: recombinant Wnt-3A, Noggin, or R-spondin1 can be substituted with conditioned medium from L-WRN (ATCC^Ⓡ^CRL-3276TM) cell line. Conditioned media: L-WRN (ATCC^Ⓡ^CRL-3276TM) cell line was cultured in DMEM supplemented with 10% FBS and 1% penicillin and streptomycin; when the cell was 70% confluent, all the media were suctioned and replaced with advanced DMEM (10% FBS, 1% penicillin and streptomycin). After washing with the media, new advanced DMEM (10% FBS, 1% penicillin and streptomycin) was added, and cultured for 2 to 3 days to obtain conditioned media.	≥5 passages of growth (P5)
Seppälä [17]	Unspecified	Unspecified	Unspecified	45	78%	Unspecified	1 mg/mL collagenase ×I, 10 μg/mL DNAse I, 10.5 μM Y-27632 in human complete medium	Advanced DMEM/F12, HEPES 10 mM, GlutaMax 1×, A83-01 500 nM, hEGF 50 ng/mL, m-Noggin 100 ng/mL, hFGF10 100 ng/mL, hGastrin I 0.01 μM, N-acetylcysteine 1.25 mM, nicotinamide 10 mM, PGE2 1 μM, B27 supplement 1× final, R-spondin1-conditioned media 10% final, and afamin–Wnt3A-conditioned media 50% final.	18–102 days
Tiriac [29]	FNB	Unspecified	Unspecified	60	72%	Unspecified	1 mg/mL collagenase ×I, 10 μg/mL DNAse I, 10.5 μM Y-27632 in human complete medium	Advanced DMEM/F12, HEPES 10 mM, GlutaMax 1×, A83-01 500 nM, hEGF 50 ng/mL, m-Noggin 100 ng/mL, hFGF10 100 ng/mL, hGastrin I 0.01 μM, N-acetylcysteine 1.25 mM, Nicotinamide 10 mM, PGE2 1 μM, B27 supplement 1× final, R-spondin1 conditioned media 10% final, and afamin–Wnt3A-conditioned media 50% final.	Not reported
Boj [9]	FNA	Unspecified	Unspecified	2	100%	Unspecified	Collagenase II (5 mg/mL, Gibco) in human complete medium. The material was further digested with TrypLE (Gibco)	Advanced DMEM/F12 supplemented with (1) GlutaMax 1×, (2) Hepes 1×, (3) Noggin 10% *v*/*v* or recombinant protein 0.1 μg/mL, (4) Gastrin 10 nM, (5) nicotinamide 10 mM, (6) R-spondin-1 10% *v*/*v*, (7) EGF 50 ng/mL, (8) FGF-10 100 ng/mL, (9) N-acetyl-L-cysteine 1 mM, (10) B27 supplement, (11) Wnt3a 50% *v*/*v*, (12) Primocin 1 mg/mL, (13) penicillin–streptomycin 1×, (14) A83-01 0.5 μΜ.	Not reported
Wiessner [30]	22G FNA/FNB	Unspecified	Head: 15; body: 30; tail: 5	50	34%	Unspecified	TrypLE	DMEM-F12 (#11320033 Thermo Fisher), 5 mg/mL D-glucose (#G8270 Sigma-Aldrich), 0.5% ITS Premix (#354350 Fisher Scientific), 5 nM 3,3,5-Triiodo-L-thyronine (#T0821 Sigma-Aldrich), 1 µM dexamethasone (#D175 Sigma-Aldrich), 100 ng/mL cholera toxin (#C9903 Sigma-Aldrich), 1% penicillin–streptomycin (#15140122 Thermo Fisher Scientific), 5% NU-Serum IV (#355500 Fisher Scientific), 25 µg/mL bovine pituitary extract (#P1167 Sigma-Aldrich), 10 mM nicotinamide (#N3376 Sigma-Aldrich), 100 µg/mL Primocin (#ant-pm05 Invivogen), 0.5 µm A83–01 (#2939 Tocris, U.K), 10% RSPO1-conditioned medium (R-spondin-1 overexpressing cell line HEK293T, provided by the Hubrecht Institute (Uppsalalaan 8, 3584 CT Utrecht, Netherlands, Netherlands), 100 ng/mL recombinant human geregulin-1 (#100–03 Peprotech), and 10 µM Rho kinase inhibitor (#TB1254-GMP Tocris).	≥5 passages of growth (P5)
Kim [31]	22G FNB	Unspecified	Head: 51; body: 31; tail: 25	113	83.2%	I: 14; II: 7; III: 33; IV: 59	Tumors were homogenized with a GentleMACS tissue dissociator and human tumor dissociation kit (Miltenyi Biotec).	Advanced DMEM/F12 supplemented with GlutaMax, B27 supplement, N2 supplement, Wnt-3a, Human R-spondin 1, human gastrin, human Noggin, human epidermal growth factor, human fibroblast growth factor 10, A83-01, Primocin, Y-27632, antibiotic antimycotics, HEPES, N-Acetylcysteine, and nicotinamide.	≥3 passages of growth (P3)
Yang [32]	22G FNA	2 times	Head: 6; body: 2; tail: 1	9	55.5%	Unspecified	Used the Advanced DMEM/F-12 medium from the American Thermo Fisher Company as the basic medium, with added collagenase ×I and dispase to prepare the digestion solution	Advanced DMEM/F-12 medium is used as the basic medium, and Wnt3A, R-Spondin, Noggin, FGF10, and other growth factors are added to form an organoid growth medium.	≥3 passages of growth (P3)
Matsumoto [33]	19G/22G FNB	Unspecified	Unspecified	27	74%	Unspecified	Liberase TH (20 min) and TrypLE Express	Advanced DMEM/F12, HEPES, penicillin–streptomycin mixed solution, GlutaMax ^TM^ (100×), afamin–Wnt3A CM, R-spondin-1 CM, EGF, Noggin, IGF-1, FGF-2, A83-01, Y-27632, B-27 supplement, N-acetyl-L-cysteine, and Gastrin I.	Not reported

## Abbreviations

**Full Name**	**Abbreviation**
Patient-derived organoids	PDOs
Endoscopic ultrasound-guided fine-needle aspiration/biopsy	EUS-FNA/FNB
Pancreatic ductal adenocarcinoma	PDAC
Patient-derived xenografts	PDXs
Rapid on-site evaluation	ROSE
Residual samples from saline flushing	RSSF
Fine-needle aspiration	FNA
Fine-needle biopsy	FNB
Hematoxylin and eosin	HE
